# Implementation of peer specialist services in VA primary care: a cluster randomized trial on the impact of external facilitation

**DOI:** 10.1186/s13012-021-01130-2

**Published:** 2021-06-07

**Authors:** Matthew Chinman, Richard Goldberg, Karin Daniels, Anjana Muralidharan, Jeffrey Smith, Sharon McCarthy, Deborah Medoff, Amanda Peeples, Lorrianne Kuykendall, Natalie Vineyard, Lan Li

**Affiliations:** 1 VISN 4 Mental Illness Research, Education and Clinical Center, VA Pittsburgh, Pittsburgh, PA USA; 2 Center for Health Equity Research and Promotion, VA Pittsburgh, Pittsburgh, PA USA; 3 RAND Corporation, Pittsburgh, PA USA; 4 VISN 5 Mental Illness Research, Education and Clinical Center (MIRECC), Baltimore, MD USA; 5 Division of Psychiatric Services Research-Department of Psychiatry, School of Medicine, University of Maryland, Baltimore, MD USA; 6 VA Behavioral Health Quality Enhancement Research Initiative (QUERI), Central Arkansas Veterans Healthcare System, North Little Rock, AR USA

**Keywords:** Peer specialist, Implementation, Facilitation, Primary care

## Abstract

**Background:**

Over 1100 veterans work in the Veterans Health Administration (VHA) as peer specialists (PSs)—those with formal training who support other veterans with similar diagnoses. A White House Executive Action mandated the pilot reassignment of VHA PSs from their usual placement in mental health to 25 primary care Patient Aligned Care Teams (PACTs) in order to broaden the provision of wellness services that can address many chronic illnesses. An evaluation of this initiative was undertaken to assess the impact of outside assistance on the deployment of PSs in PACTs, as implementation support is often needed to prevent challenges commonly experienced when first deploying PSs in new settings.

**Methods:**

This study was a cluster-randomized hybrid II effectiveness-implementation trial to test the impact of minimal implementation support vs. facilitated implementation on the deployment of VHA PSs in PACT over 2 years. Twenty-five Veterans Affairs Medical Centers (VAMCs) were recruited to reassign mental health PSs to provide wellness-oriented care in PACT. Sites in three successive cohorts (*n* = 7, 10, 8) over 6-month blocks were matched and randomized to each study condition. In facilitated implementation, an outside expert worked with site stakeholders through a site visit and regular calls, and provided performance data to guide the planning and address challenges. Minimal implementation sites received a webinar and access to the VHA Office of Mental Health Services work group. The two conditions were compared on PS workload data and veteran measures of activation, satisfaction, and functioning. Qualitative interviews collected information on perceived usefulness of the PS services.

**Results:**

In the first year, sites that received facilitation had higher numbers of unique veterans served and a higher number of PS visits, although the groups did not differ after the second year. Also, sites receiving external facilitation started delivering PS services more quickly than minimal support sites. All sites in the external facilitation condition continued in the pilot into the second year, whereas two of the sites in the minimal assistance condition dropped out after the first year. There were no differences between groups on veterans’ outcomes—activation, satisfaction, and functioning. Most veterans were very positive about the help they received as evidenced in the qualitative interviews.

**Discussion:**

These findings demonstrate that external facilitation can be effective in supporting the implementation of PSs in primary care settings. The lack of significant differences across conditions after the second year highlights the positive outcomes associated with active facilitation, while also raising the important question of whether longer-term success may require some level of ongoing facilitation and implementation support.

**Trial registration:**

This project is registered at ClinicalTrials.gov with number NCT02732600 (URL: https://clinicaltrials.gov/ct2/show/NCT02732600).

Contributions to the literature

- The model of facilitation used in the study can be a model of deploying peer specialists into primary care in other sites.
- The model of facilitation used in the study can also be a model of deploying new workers (not just peer specialists) into a variety of health care settings.
- Given there were no funds to hire new peer specialists, the study shows some level of ongoing facilitation may be needed to support peer specialist implementation.

## Background

Over 1100 veterans work in the Veterans Health Administration (VHA) as peer specialists (PSs). PSs are veterans with lived mental health experience and formal training who provide support to other veterans with similar diagnoses. Drawing on their own recovery experience, PSs provide a range of services including facilitating groups, role modeling, providing outreach and support, teaching coping skills, and helping veterans navigate and coordinate care. PSs have traditionally supported recovery from mental illness and substance use disorders in mental health settings. While not conclusive, randomized and quasi-experimental trials have shown that individuals with mental illnesses who receive PS services benefit across a range of outcomes, such as improved functioning and patient activation [1].

PSs are increasingly being deployed beyond mental health treatment settings to support holistic physical health and wellness in other medical settings such as primary care. In 2014, the White House issued an Executive Action mandating the pilot reassignment of VHA PSs from mental health to 25 primary care Patient Aligned Care Teams (PACTs) in order to extend delivery of peer support to veterans in primary care settings. VHA PACTs, first established in 2010, are medical homes that provide coordinated, comprehensive patient-centered outpatient primary care using a multidisciplinary team-based approach [2]. Models like VHA PACT, especially those that use quality improvement methods, have been shown to be effective in managing chronic conditions [3–5]. Importantly, to maximize the effectiveness of this type of coordinated, team-based, complementary support, “patient activation” is often needed [6–8], which is the knowledge, skills, and motivation to be involved in one’s own care [9, 10]. One such intervention which can increase patient activation is health coaching, a patient-centered approach that includes assessing needs, developing concrete goals, and skill building [6, 11–15]. Research also indicates that such supportive, complementary coaching interventions delivered by peers, or individuals who share a common characteristic with a target population, can make primary care services more effective by activating patients to engage in more health behaviors such as illness self-management, disease prevention, and health information seeking [16, 17].

Although PS services are expanding from mental health to other medical settings, there has been no research to examine how best to implement PS services in primary care settings. However, within mental health settings, there have been many documented challenges to the implementation of PS services, especially when these services were just starting. For example, lack of understanding regarding the PS role, inadequate supervision, lack of training opportunities, stigma, and discrimination against PSs have been common implementation challenges [18–27], all of which can lead to poor uptake of PS services. One strategy that has been examined to implement PS services in mental health settings is external facilitation [19]. External facilitation is an evidence-based implementation strategy specified within the integrated Promoting Action on Research Implementation in Health Services (i-PARIHS) framework [28–32], in which outside individuals activate and support implementation by assessing and responding to characteristics of recipients of the innovation, taking into consideration additional factors within their inner organizational and outer contextual settings [28]. It is a multifaceted strategy involving interactive problem-solving and support that occurs in a context of a recognized need for improvement and supportive interpersonal relationships [33]. Several studies have shown external facilitation to be an effective strategy for improving implementation of complex evidence-based practices and other clinical innovations [30, 34–37], as well as improving patient-level outcomes [38–41]. Thus, we hypothesized that facilitation would help sites and their PSs overcome multiple challenges and have better implementation, which would then lead to better outcomes of patient activation, satisfaction with overall care, and self-rated health. As detailed below, the purpose of this study is to evaluate the impact of external facilitation on implementing PS services and veteran outcomes in VA primary care settings.

## Methods

The present study evaluates a pilot project to deploy PSs in 25 VA primary care settings. It is a hybrid II, cluster randomized trial comparing 12 VA medical centers (VAMCs) receiving external facilitation to 13 VAMCs receiving minimal assistance. Including PSs in PACT teams were intended to extend the provision of VA PS services beyond mental health settings and could include some form of brief health coaching. Sites agreed to participate in the pilot for 2 years. To facilitate health coaching, VA Central Office (VACO) made whole health coach training available for most PSs engaged in this pilot. As is common in hybrid type II designs [42], the project has a dual emphasis on the assessment of both uptake of PS services and veteran-level outcomes. Key outcomes for this evaluation were specified according to the RE-AIM model [43], an evaluation model that specifies five domains needed for successful impact of a new intervention (i.e., *R*each, *E*ffectiveness, *A*doption, *I*mplementation, and *M*aintenance). The present manuscript presents findings relating to Reach and Adoption as measured using administrative services data. We also report on the Effectiveness of PS-delivered services on veteran satisfaction, activation, and functioning. Qualitative interviews with 10 veterans in receipt of PS services (6 in the external facilitation sites and 4 in sites receiving minimal implementation assistance) were also conducted to characterize and describe participant experiences regarding the PSs’ Effectiveness. Finally, we also report on the maintenance (sustainment) of PS-delivered services beyond the first and second years specified in the pilot.

In collaboration with our VA national operational partners, the project was determined to be quality improvement by the VA, thus individual informed consent for the peer specialists, non-peer providers and supervisors, and Veterans was not required or sought. However, veterans are always free to decline PS and participation in the evaluation. Full details of this process are available elsewhere [44]. Facilitators and research staff watched for harms of PSs while they were active. None were reported.

### Site recruitment and randomization

A convenience sample of 25 PACT sites were recruited by a workgroup of national VA leaders led by the VA’s National Director of Peer Support and National Director of Integrated Services and the project team. To participate, sites demonstrated endorsement from facility leadership and had to agree to dedicate one or more already-hired PSs to PACT for a total of 10 or more hours per week, for 1 year. No additional funds were made available, thus sites had to reassign their current PSs deployed in mental health settings, in part or in full, to work in PACT. The actual amount of PS time dedicated to PACT across sites ranged from 4 h per week (in one site) up to 80 h per week covered by two or more PSs (in 3 sites). Most PSs worked 10 h a week (in 8 sites) or 20-50 h a week (in 13 sites).

From January 2016 to March 2019, 25 recruited sites were divided into three cohorts. A new cohort was introduced over three successive 6-month blocks. Our original plan was to establish three successive cohorts of *n* = 8, *n* = 8, and *n* = 9 sites. In cohort 1, 8 sites were pair-matched based on the number of PSs deployed at the site, number of hours each PS would provide in PACT, and the status of PS assignment to PACT (currently deployed, *n* = 2, or anticipating deployment to PACT as part of the pilot, *n* = 23). A statistician with no other direct involvement in the project used a computerized random number generator to assign sites within each pair to 1 year of either minimal implementation support (*n* = 4) or external facilitation (*n* = 4). A control site in cohort 1 left the study early on, prior to when any data collection or any study procedures were implemented. Because of the uneven number of sites, randomization in cohort 2 pair-matched 10 sites, randomly assigned an entire pair to the minimal implementation support condition, and then proceeded to assign the remaining sites, within each pair, to either facilitation (*n* = 4) or minimal implementation support (*n* = 6). Finally, sites in cohort 3 were pair-matched and within each pair, randomly assigned to either facilitation (*n* = 4) or minimal implementation support (*n* = 4). Across all cohorts, the total number of sites receiving facilitation was 12, and the total number of sites receiving only minimal implementation support was 13. Blinding to condition assignment was not possible because it was well known who received the external facilitation. Across sites, there was a total of 43 PSs and 44 supervisors. The average age of the PSs was 52, 81% were male, 52% Caucasian, and 48% African-American or other race, and the mean number of years spent working in the VA was 3.9 (s.d. = 2.3). There were no differences among PSs in age, gender, and race across conditions. There was, however, a significant difference on the mean number of years PSs had spent working in the VA between external facilitation (3.0 ± 1.5) and minimal implementation support sites (5.1 ± 2.7), *p* = 0.007.

### External facilitation versus minimal implementation support

External facilitation, provided for 1 year at each site by one of three doctorate level psychologists, was tailored to site needs and adapted to incorporate lessons learned about the implementation challenges experienced when first deploying peer specialists [45]. Facilitation was organized across two phases: pre-implementation (i.e., preparatory tasks) and implementation (i.e., monitoring and troubleshooting). As is typical for external facilitation, facilitators engaged in multiple proactive strategies, tailored to each location [46]. These activities are summarized in Table 1 [29, 42, 46–48].

**Table 1:** Facilitation activities compared to activities in the minimal assistance condition [44]

Stage	Facilitation	Minimal assistance
Pre-implementation	•Conduct a webinar with site stakeholders about mental health/primary care collaboration •Provide VA’s PS implementation toolkit •Conduct implementation needs assessment with key stakeholders •Conduct 2-day site visit •Clarify purpose/role of facilitation staff; share organizational assessment data; set expectations •Assess and engage facility stakeholders who will be impacted by the implementation •Educate PACT on implementation strategies and/or PS evidence •Assist PACT in developing goals for assessing progress (critical tasks, persons responsible, PACT needs) modified for the project •Address needs for local customization prior to implementation	•Conduct a webinar with site stakeholders about mental health/primary care collaboration •Provide VA’s PS implementation toolkit
Implementation	•Option to call VHA Central Office staff overseeing the pilot for ad hoc consultation •Monthly Learning Collaborative calls were held for sites (one for PSs and one for PSs’ supervisors) •Biweekly calls to discuss status of implementation and problem-solving as needed •Monthly calls to include all facilitation sites (within each cohort) to facilitate information sharing •Accessibility to stakeholders by telephone/email for additional support or consultation as needed •Monitor and provide feedback on progress in achieving implementation goals/milestones •Aid problem-solving by leveraging local resources, sharing solutions, or identifying VA resources •Monitor use and impact of identified solutions for problems/barriers	•Option to call VHA Central Office staff overseeing the pilot for ad hoc consultation •Monthly Learning Collaborative calls were held for sites (one for PSs and one for PSs’ supervisors)

Facilitators began this process by conducting telephone conference calls with local site team members to assess a range of domains related to the local context—e.g., available resources for PS implementation—using a standardized semi-structured interview guide. These site assessment calls were followed by an in-person, two-day site visit to connect with key staff, and complete a PS implementation checklist to develop a specific implementation plan tailored to each site. These site visits also provided opportunities for the external facilitators to further develop relationships with site stakeholders, provide staff and leadership with an overview of the project and the PS role, and build consensus. Once the local implementation plan had been finalized, the facilitators then engaged the local implementation team, including PSs and supervisors, in bi-weekly phone or Skype meetings to monitor and support implementation of the plan and encourage accountability. These meetings frequently involved feedback of PS administrative services data extracted from the VA electronic medical record.

While the overall structure of the facilitation did not change from cohort to cohort, we did refine our methods over time. For example, over time, we were better able to pull PS service delivery data and feed it back to sites for use in quality improvement purposes. As we learned more about the specific challenges facing sites, we were able to develop new educational materials (e.g., a document entitled, “What can Peers do?”). Also, we refined the implementation checklist and we referred PSs and supervisors to consult with sites from earlier cohorts.

The role of the facilitators should be contrasted with the PS supervisors. Each site decided who each PS’s supervisor was going to be. Supervisors met with PSs to discuss individual veterans and their care; the external facilitators met with the supervisors, PSs, and other primary care stakeholders about incorporating PSs into the primary care work flow. External facilitators did strongly recommend to each site that PSs receive regular supervision, and inquired about whether it was happening in the regular facilitation meetings.

The average number of hours of external facilitation (operationalized as direct contact between facilitator and sites) ranged from 5.98 to 37.25 h with an average of 23.26 (sd = 9.77) hours across the 12 sites receiving this implementation support. Each site received about 1 h per month of faciliation, although the external facilitators also spent time helping sites outside of site meetings (e.g., reviewing data, preparing documents, etc.). More details about the external facilitation strategy applied in this project are available elsewhere [44].

The assistance offered to the minimal implementation support sites included written guidance (a toolkit on how to hire and integrate PSs into a clinical setting), a 1-h webinar on integrating mental health and primary care, and the option to call VHA Central Office staff overseeing the pilot for ad hoc consultation. Further, two separate monthly learning collaborative calls were held for sites within each of the two study conditions; one for PSs and one for PSs’ supervisors. Facilitated by a consultant expert in PS services, these calls afforded staff from all sites the opportunity to meet together to review implementation progress, share ideas and lessons learned, and provide support.

### Peer specialist services delivered

PSs provided a variety of services across the sites, through a mix of phone and in-person visits. Approximately 30 of the 43 PSs across sites received training in Whole Health Coaching (WHC) from the VA Office of Patient-Centered Care and Cultural Transformation, and incorporated aspects of the training into their activities with veterans, including the completion of personalized health plans and individual or group-based WHC. An overarching goal for PSs utilizing WHC was to help veterans identify realistic goals, and then provide or connect veterans with the support needed to achieve those goals. Identifying and connecting veterans with VA and community resources was another important service provided. PSs also facilitated groups, independently or with another co-leader from PACT, on topics ranging from dental education and smoking cessation to PTSD support and chronic disease self-management.

### Measures and analytic methods

In addition to the measures below, the study initially had planned to collect data on the organizational readiness and level of team cohesion of the groups the PSs had joined in primary care (both designated as secondary outcomes in the Clinical Trials record for this study). This plan assumed that each site would have a cohesive team and that information about the team would be useful in interpreting other study data. However, the teams the PSs joined were loose affiliations and thus the data from those assessments were not useful. Results from this trial are available at https://clinicaltrials.gov/ct2/show/record/NCT02732600. In that study record, outcomes that addressed provider behavior or patient outcomes were designated as primary or secondary. Information about how to obtain each measure is available from the first author.

#### Reach and adoption

Two years of biweekly administrative data pulls from the VA Corporate Data Warehouse were used to assess measures of Reach and Adoption of PS services from the RE-AIM framework. Reach was operationalized as: (1) the number of unique Veterans who received services from PSs, and (2) the average number of visits per Veteran. Adoption was operationalized as: (1) the total number of services provided by each PS; and (2) each site’s time to first PS service delivered from the start of the cohort, as an indirect measure of the site’s ability to get their program operational.

These variables were calculated as both “raw” scores—the total number of unique veterans and services in the 2-year period for each PS regardless of the PS employment period and hours worked and as “adjusted” scores. The adjusted workload variables took into consideration both the employment period (many PSs did not start immediately or may have left prior to the end of the 2 years) and weekly hours worked (varying from 1 h to 40 h per week). Visits during each PS’s employment period were divided by the total number of hours worked, then multiplied by 40 to calculate adjusted values for both operationalizations of Reach (as defined above) and total number of services (Adoption) provided per a 40-h work week. Because these variables were significantly skewed, we used a log transformation to improve their distributional properties. Differences between intervention conditions were then compared with a series of analyses of covariance models with age, gender, and race as covariates. Since these variables are measured at the PS level, the covariates were the average across the veterans seen by each PS (mean age, percent White, and percent male). The average number of visits per veteran was also compared across conditions. This is a veteran-level variable, thus a General Linear Mixed Model (GLMM) was used with PS specified as a random effect and veteran age, race, and gender included as covariates.

Before comparing conditions, we removed two sites from the first cohort that were matched with each other, both of which already had PSs working in PACT (one in each condition) at the time of joining the pilot. These sites had substantially more PS services provided than all other sites and their inclusion initially obscured the results for the rest of the sites. Thus, we present raw totals from the 25 sites, but focused on the remaining 23 sites in this pilot that were newly implementing PS services in primary care in the adjusted comparisons (as defined above).

#### Effectiveness

**Fig. 1:**
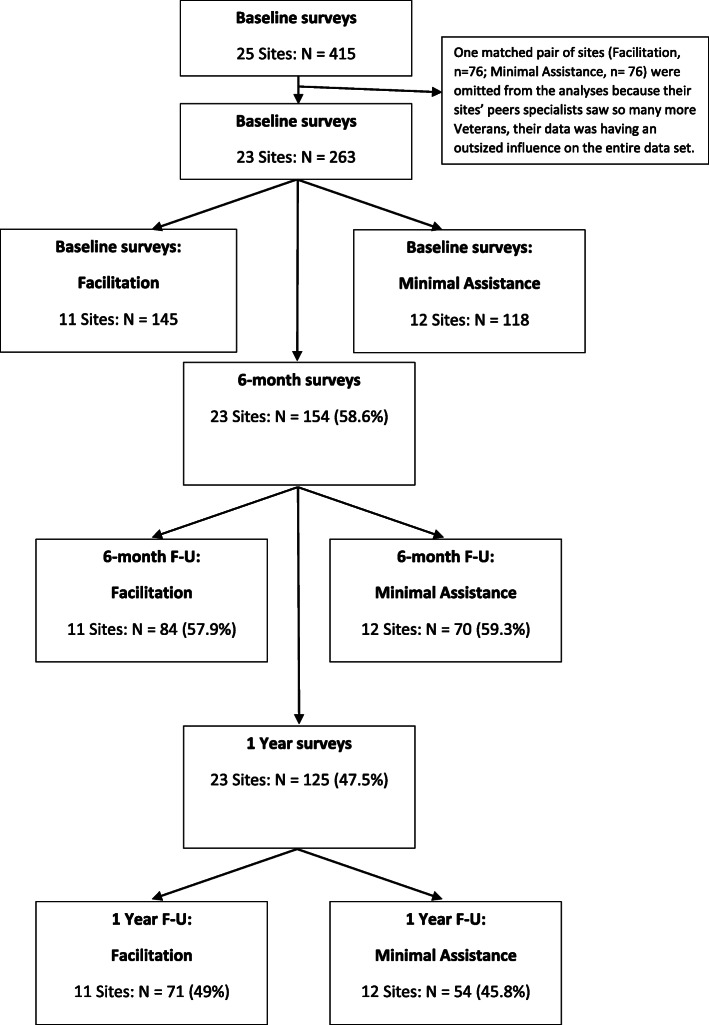
Study flow diagram

PSs asked veterans to complete a brief survey during one of their first interactions with the veteran. Project evaluation staff collected follow-up assessments at 6-months (timepoint 2, TP2) and 1-year (timepoint 3, TP3) by phone or mail. A total of 415 baselines were completed across the 25 participating sites. For the 23 sites used for all between condition comparisons, a total of 263 baseline surveys were completed; a greater proportion of veterans from the facilitation sites completed baseline surveys than did veterans from minimal implementation support sites (Chi-square 6.42; *p* = 0.011). Of these 263 baselines, 154 (59%) completed TP2 assessments and 125 (48%) completed TP3 assessments (see Fig. 1). There were no differences in response rates across study conditions (*p* = 0.820 at TP2, *p* = 0.605 at TP3).

Three measures were included in the veteran level assessment completed at each timepoint: (1) a single item regarding general health functioning taken from the VR-12, a veteran version of the SF-36 Health Inventory [49–51]; (2) the Patient Activation Measure (PAM—the primary outcome listed in the clinical trials record for this study), a 13-item survey that measures an individual’s perceived ability to manage his or her illness and health behaviors and act as an effective patient [10]; and, (3) a modified version of the 12-item Satisfaction Index-Mental Health Survey (designated as a secondary outcome in the clinical trials record for this study) [52]. A General Linear Mixed Model (SAS v 9.4 Proc MIXED) was used to compare conditions over time (baseline, 6 months, 12 months) with veteran age, race, and gender as covariates and site as a random effect. ICCs ranged from .001 to .07. All available data were used at each timepoint.

As a measure of Effectiveness, semi-structured, qualitative interviews were also completed via telephone with a total of 10 veterans (3 female and 7 male) across the three cohorts to characterize and describe participant experiences working with PSs in PACT settings. These veterans were identified as having had a high number of documented contacts with the PS (range = 8-34). Interviews were professionally transcribed. A senior member of the evaluation team read the interview transcripts and summarized veterans’ experiences with and reactions to working with PSs. A second member of the team who had also read the transcripts then reviewed the summary for accuracy.

## Results

### Sample demographics

Across all 25 participating sites, the demographics of the full sample are presented in rows one through three in Table 2; for the 23 sites that newly implemented PSs in PACT (used in the adjusted Reach and Adoption analyses), demographics are presented in rows four through six. Finally, demographics across all 23 sites for participants who completed surveys for our Effectiveness measures are presented in rows seven through nine.

**Table 2:** Demographics

								Comparison			
Label		Total		External facilitation		Minimal implementation support		Test	df	Test value	p
**Full sample, unadjusted**^**1**^		**n**	**(*N*** **= 5616)**	**n**	**(*N*** **= 2091)**	**n**	**(*N*** **= 3525)**				
Mean age		5554	61.5 ± 13.9	2062	60.6 ± 13.3	3492	62.0 ± 14.2	t	4545	−3.78	< 0.0001
Gender, %	Female	5554	649 (11.7%)	2062	338 (16.4%)	3492	311 (8.9%)	Chi-sq	1	70.40	< 0.0001
	Male		4,905 (88.3%)		1724 (83.6%)		3181 (91.1%)		.	.	.
Race/ethnicity, %	1 = Non-His. White	5228	4042 (77.3%)	1930	1158 (60.0%)	3298	2884 (87.4%)	Chi-sq	2	530.51	< 0.0001
	2 = African American		978 (18.7%)		654 (33.9%)		324 (9.8%)		.	.	.
	3 = Other		208 (4.0%)		118 (6.1%)		90 (2.7%)		.	.	.
**Sample adjusted reach and adoption data**^**2**^		**n**	**(*N*** **= 2383)**	**n**	**(*N*** **= 1484)**	**n**	**(*N*** **= 899)**				
Mean age		2353	57.9 ± 14.3	1464	58.6 ± 14.1	889	56.8 ± 14.7	t	2351	3.04	0.002
Gender, %	Female	2353	426 (18.1%)	1464	272 (18.6%)	889	154 (17.3%)	Chi-sq	1	0.59	0.443
	Male		1927 (81.9%)		1192 (81.4%)		735 (82.7%)		.	.	.
Race/ethnicity, %	1 = Non-His. White	2118	1291 (61.0%)	1336	821 (61.5%)	782	470 (60.1%)	Chi-sq	2	4.64	0.098
	2 = African American		670 (31.6%)		406 (30.4%)		264 (33.8%)		.	.	.
	3 = Other		157 (7.4%)		109 (8.2%)		48 (6.1%)		.	.	.
**Veteran survey/effectiveness data**^**3**^		**n**	**(*N*** **= 263)**	**n**	**(*N*** **= 145)**	**n**	**(*N*** **= 118)**				
Mean age		225	58.6 ± 13.6	135	57.4 ± 14.8	90	60.5 ± 11.3	t	219	−1.78	0.076
Gender, %	Female	226	35 (15.5%)	135	15 (11.1%)	91	20 (22.0%)	Chi-sq	1	4.90	0.027
	Male		191 (84.5%)		120 (88.9%)		71 (78.0%)				
Race/ethnicity, %	1 = Non-His. White	210	133 (63.3%)	124	85 (68.5%)	86	48 (55.8%)	Chi-sq	2	4.22	0.121
	2 = African American		62 (29.5%)		30 (24.2%)		32 (37.2%)				
	3 = Other		15 (7.1%)		9 (7.3%)		6 (7.0%)				

^**1**^Demographics of all those who received services at the 25 participating sites

^**2**^Demographics of all those who received services the 23 sites that newly implemented PSs in PACT (used in the adjusted Reach and Adoption analyses)

^**3**^Demographics across all 23 sites for participants who completed surveys for the Effectiveness measures

### Reach

Using raw numbers, 3841 unique veterans were reached across all 25 sites during the first year (during which external facilitation was provided to the experimental condition sites) and 5616 unique veterans were reached across both years. The average number of visits per veteran across all 25 sites was 1.9 during the first year and 2.3 across both years.

As noted above, before looking at comparisons across conditions, we removed two matched sites who already had PSs working in PACT (one in each condition) and used adjusted comparisons (as defined above) to focus more specifically on sites that were newly implementing PSs in primary care. Compared to minimal assistance sites, sites receiving external facilitation saw a significantly larger average number of unique veterans (*p* = 0.046) at the end of the first year; though across both years there was no significant difference between conditions (*p* = 0.428). Though the average number of PS visits per veteran trended higher for sites receiving external facilitation, these differences were not significant for the first year (*p* = 0.419) nor across both years (*p* = 0.266). Reach data are presented in Table 3.

**Table 3:** Adjusted measures of Reach and Adoption among 23 sites newly implementing peer support in primary care

Reach	Per service week in actual date range, Means ± SD^**1**^			Test	Comparison		
	Total	External facilitation	Minimal implementation support		df	Test value	p
**Unique veterans**							
First year only	3.2 ± 4.9	4.8 ± 6.1	1.3 ± 1.3	t	31	2.10	0.046
Across both years	5.9 ± 7.0	6.0 ± 5.8	5.8 ± 8.5	t	34	0.80	0.428
**Visits per veteran per PS**							
First year only	2.4 ± 3.1	2.6 ± 3.6	2.0 ± 2.1	t	1053	0.81	0.419
Across both years	2.8 ± 4.8	3.2 ± 5.6	2.2 ± 3.0	t	2058	1.11	0.267
**Adoption**							
**Total visits**							
First year only	5.9 ± 7.2	8.4 ± 8.7	2.7 ± 2.7	t	31	2.64	0.014
Across both years	11.2 ± 8.7	12.4 ± 8.7	9.8 ± 8.7	t	34	0.98	0.336
**Time to service**							
# of days from cohort start date to first PS visit date	140.6 ± 110.1	88.7 ± 65.0	188.2 ± 123.4	t	21	−2.38	0.027

^**1**^Workload variables adjusted for the employment period and weekly hours worked (1-40). Visits during each PS’s employment period were divided by the total number of hours worked, then multiplied by 40 to calculate adjusted values for both operationalizations of Reach and total number of services (Adoption) provided per a 40-h work week

### Adoption

Using raw unadjusted numbers, a total of 7153 visits were held across all 25 sites during the first year, and 12,771 were held across both years. Within the 23 sites where PSs were newly added to PACT and using adjusted data (as defined above), external facilitation sites had a significantly greater average number of total visits in the first year than did minimal support sites (*p* = 0.014); though across both years there was no significant difference between conditions (*p* = 0.336) (see Table 3). Also, within the 23 sites, newly implementing PSs, time to first service was significantly shorter in sites receiving external facilitation (89 days) than in sites receiving minimal implementation support (188 days), *p* = 0.027.

### Effectiveness

#### Quantitative measures

As shown in Table 4, there were no significant group by time effects at between baseline and 6 months or baseline and 1 year on any of the veteran outcome measures across the 23 sites (results were the same when analyzing data from the 25 sites).

**Table 4:** Effectiveness outcomes for veterans served by peer specialists

	Baseline, M ± SD		Month 6 follow-up, M ± SD		Baseline-6 month comparison				Year 1 follow-up, M ± SD		Baseline-1 year comparison			
	External facilitation	Minimal support	External facilitation	Minimal support					External facilitation	Minimal support				
Effectiveness measure	(***N*** = 145)	(***N*** = 118)	(***N*** = 84)	(***N*** = 70)	ES	t	df	p	(***N*** = 71)	(***N*** = 54)	ES	t	df	p
In general, would you say your health (0-100)^1^	49.5 ± 27.8	47.4 ± 26.7	46.5 ± 29.6	49.1 ± 29.3	−2.51	−0.46	188	0.644	44.9 ± 26.6	45.7 ± 23.5	−1.70	−0.37	188	0.711
Patient satisfaction total score (12-72)	50.3 ± 12.1	46.4 ± 12.7	51.7 ± 13.0	49.2 ± 13.8	−1.21	−0.50	207	0.616	51.2 ± 14.4	49.1 ± 13.9	−1.91	−0.64	207	0.522
PAM total score (13-52)	40.9 ± 6.4	40.5 ± 5.6	40.7 ± 5.8	40.1 ± 6.2	0.07	0.08	205	0.940	40.7 ± 5.2	39.0 ± 6.7	1.75	1.43	205	0.154
PAM patient activation level (0-100)	62.6 ± 16.2	61.2 ± 15.5	61.8 ± 15.5	60.4 ± 17.9	−0.85	−0.35	205	0.724	61.6 ± 14.5	57.6 ± 16.6	3.57	1.30	205	0.196

Note: Raw means and Proc Mixed repeated measurement analyses are presented for continuous data with condition and time interaction

^1^General health rating has five levels: 100 = Excellent; 99-85 = Very good; 84-60 = Good; 59-35 = Fair; 34-0 = Poor

#### Qualitative findings

Most veterans who were interviewed (*n* = 10) were very positive about their experiences working with a PS. They described PSs as being “terrific,” “professional,” “easy to talk to,” and “knowledgeable.” Veterans noted that the PS helped them in meeting their personal goals (e.g., diabetes management and weight loss) with a positive attitude and nonjudgmental accountability. Some veterans said that the PS also helped them get connected with other services and programs, both within the VA and in the community. PSs functioned not only as a sounding board (“he’s the only one that listens to me”) but also as a liaison between Veterans and their PACT teams and other providers (“he knew how to get things done”). PSs’ veteran status was “the key thing” for many veterans because it helped establish an immediate comradery: “it’s different if you’ve been there and done that or if you just learned it off a book.” Notably, several veterans said that working with a PS gave them a better view of the VA overall, with one veteran saying that the experience “restored my faith and relationship with the VA.” A few of the veterans also expressed negative sentiments about working with a PS; these complaints centered on aspects of program delivery (e.g., the focus on a particular model of Whole Health coaching rather than open-ended peer support, difficulty getting appointments with the PS). Still, even the least-satisfied veteran said that the program was “better than nothing” and that he found it helpful “knowing somebody out there was interested in helping you get better.”

#### Maintenance

We also tracked the number of sites across study conditions that continued using PSs in PACT after the first and second years. After the first year, all external facilitation sites (12 out of 12) continued into the second year; two of the minimal support sites discontinued (11 out of 13 continued). Regarding continuation beyond 2 years, there was not a difference in rates of continuation by condition; fourteen (56%) of the twenty-five sites were able to sustain the PS’ presence (6 external facilitation, 8 minimal support) and 11 discontinued (6 external facilitation, 5 minimal support) after the project period (Chi-square = 0.561, *p* = 0.34).

## Discussion

In the first year, sites that received external facilitation had higher numbers of unique veterans served (operationalized as Reach according to the RE-AIM framework) and a higher number of visits (operationalized as Adoption). Also, sites receiving external facilitation were able to start delivering PS services more quickly than comparison sites receiving minimal implementation support. Finally, all sites in the external facilitation condition continued in the pilot into the second year, whereas two of the sites in the minimal assistance condition dropped out after the first year. These findings demonstrate that external facilitation can be effective in supporting the implementation of PSs in primary care settings, as it has been in mental health settings [19].

The lack of significant differences across conditions after the second year—in terms of service delivery and sustainability (maintenance in RE-AIM)—highlights the positive outcomes associated with *active* facilitation, while also raising the important question of whether longer-term success may require some level of ongoing facilitation and implementation support. Implementation research in other fields has documented a drop off in implementation after active implementation support ended [53]. In another study focused on integrating new mental health providers into primary care settings, a facilitation was applied for up to 28 months at participating sites to produce and sustain significant improvements in RE-AIM measures of Reach and Adoption [30, 54]. Thus, a combination of data from this and other implementation studies suggest incorporating new types of providers into primary care involving significant provider engagement and education, new roles for clinical staff, and new clinical processes and referral patterns may require the use of more enduring facilitation strategies to produce sustained improvements in service delivery.

Regarding Effectiveness, the qualitative interviews did show that veterans generally reported positive experiences and outcomes in working with PSs. However, there were no group differences in veteran-level outcomes of satisfaction, activation, or functioning. Our hypothesis that facilitation could improve services, which could then lead to better outcomes, was not supported. Research on other PS initiatives has involved much more intensive interventions than what was delivered here (average of 2.3 services to each veteran). It is likely, for example, that a total of two or three visits—which is not atypical for health coaching in primary care—may be inadequate to meaningfully impact these particular outcomes. Additionally, the lack of significant effects may also be due to timing of assessments which may have been months after the Veteran had contact with PSs. There was also great variability in the PS services delivered and the target of those services, and the limited outcome measures that were collected may not have been sensitive enough to detect that wide variety of outcomes. Other unmeasured outcomes such as recovery, hope, and resiliency—measures that have shown improvement in other PS studies (e.g., [55, 56])—may have been more impacted by working with a PS. As such, future studies that more narrowly specify the content of the PS intervention and conduct appropriately timed and more targeted assessments are needed to better understand the impact of PS services in this setting.

Given the recent passage of the Mission Act [57], which in part mandates the hiring and integration of PSs in 30 primary care settings across the VHA, the results of this study have several implications for how to improve the effectiveness of external facilitation designed to support PSs in primary care. First, the number and types of people charged to work with external facilitators should be considered; e.g., perhaps an internal facilitator might be added at the local level to supplement and extend the external facilitator’s efforts, and the duration of active facilitation may need to be extended. Second, perceived need for assistance, or other site-level idiosyncrasies (including available money and resources to invest) may also impact which types of implementation support are required (or feasible) to help sites adopt and sustain new models of care delivery. Future studies might consider adding assessments to characterize these elements including measures that look at the extent to which program goals and objectives have been written into policies and standard operating procedures (SOPs, including job descriptions), the allocation/assignment of permanent staff positions and protected space as additional metrics of adoption and institutionalization of care delivery models [58]. Third, the external facilitators in the present study are individuals who are very familiar with PSs’ training and role, which may be an important requirement for facilitators in future PS implementation efforts. Fourth, our study only used one specific type of implementation support (i.e., external facilitation). It may be that impacts on service delivery and patient outcomes may be enhanced by using alternative or multiple types of implementation support. For example, as noted above, external facilitation could be used to train internal facilitators and site champions to take a longer-term lead in integrating new providers into the primary care setting and institutionalizing the programmatic change [30]. Finally, PSs are increasingly being used not only in mental health settings but also to help address a range of health behaviors such as weight management, management of chronic health conditions [59], and addictions [60]. Given the challenges faced by sites when PSs are first introduced, including lack of role clarity and inadequate supervision, it would be advantageous to adopt a similar set of implementation strategies that were used in this effort in future PS deployment efforts.

### Limitations

The present study had some limitations that should be noted. PSs typically had limited time in primary care, some as few as 4 h a week. Further, the PSs were “on loan” from each site’s mental health service, which likely impacted their ability to deliver services during the pilot, and perhaps influenced their sustainability. In addition, there was a lack of standardization of what interventions PSs provided (PSs tailored their services to their individual settings); more information is needed on what PS interventions are effective in primary care and in what dose. In addition, the target veteran group differed by site, and sites also differed in terms of how and where they received referrals. Baseline veteran-level data was collected by the peer specialists themselves, which may have biased responses. Some peer specialists were reluctant or uncomfortable asking veterans to complete the survey, and this reduced the available veteran data. The present study also did not examine the impact of PS services on service use outcomes outside of primary care; future studies could examine whether PS support helps to improve use of health care services (as has been done in other studies, (e.g., [61–63]) and reduce use of high-cost services. Also, the study relied on self-reported measures of effectiveness. Lastly, the extraction and analysis of administrative data may not have accurately captured all the services delivered by PSs. Utilizing the reporting structure within each local sites’ electronic health record was challenging because PSs occasionally did not record their visits and coding of the visits would change without our knowledge.

## Conclusions

It is notable that external facilitation had success given that implementing PS services in a new setting like primary care requires in-depth education of providers and leadership regarding an entirely new discipline. With only a modest investment of external facilitation resources, sites that received implementation support were able to deploy PSs more quickly and deliver more services than sites without such support. This approach could be a model to other PS initiatives such as the MISSION Act.

## Data Availability

Readers can request data and materials from the lead author.

## Abbreviations

i-PARIHS: Integrated Promoting Action on Research Implementation in Health Services
PACT: Patient Aligned Care Teams
PSs: Peer specialists
VAMC: Veterans Affairs Medical Centers
VHA: Veterans Health Administration
